# Draft Genome Sequences of Two Streptococcus pneumoniae Strains Causing Invasive Infections in Children in Qatar

**DOI:** 10.1128/MRA.00511-19

**Published:** 2019-06-06

**Authors:** Clement K. M. Tsui, Sathyavathi Sundararaju, Hassan Al Mana, Eva Thomas, Patrick Tang, Mohammad Rubayet Hasan, Andres Perez-Lopez

**Affiliations:** a Department of Pathology, Sidra Medicine, Doha, Qatar; b Department of Pathology and Laboratory Medicine, Weill Cornell Medicine—Qatar, Doha, Qatar; University of Rochester School of Medicine and Dentistry

## Abstract

Invasive pneumococcal infections are a major cause of morbidity and mortality in the pediatric population. We report the draft genomes of two clinical Streptococcus pneumoniae isolates associated with severe infections in children in Qatar. The genome statistics are described, along with the strain types and serotypes predicted from the assembled genomes.

## Abstract (precis)

Invasive pneumococcal infections are a major cause of morbidity and mortality in the pediatric population. We report the draft genomes of two clinical Streptococcus pneumoniae isolates associated with severe infections in children in Qatar.

## ANNOUNCEMENT

Streptococcus pneumoniae is the leading cause of invasive bacterial infections in the pediatric population worldwide, with a wide spectrum of clinical syndromes ranging from occult bacteremia to meningitis (1, 2). The increase in antimicrobial resistance in invasive pneumococcal disease (IPD), particularly to β-lactam antibiotics and macrolides, has become a major public health concern (3). The advent of the 7-valent and 13-valent pneumococcal conjugate vaccines (PCV-7 and PCV-13, respectively) has led to a dramatic decrease in the incidence of IPD caused by serotypes included in vaccine formulations in children (1). However, an increase in the incidence of severe forms of IPD caused by nonvaccine serotypes has been reported (4). On the other hand, it has also been speculated that the rise of IPD caused by certain nonvaccine serotypes may have been driven by capsular switching and serotype replacement among some pneumococcal genotypes (5, 6). For example, the increased proportion of IPD caused by serotype 19A after the introduction of PCV-7 could be attributed to capsular switching in sequence types (STs) which were previously associated with vaccine serotype 4 (7). PCV-7 was introduced in the routine immunization program in children in Qatar in 2005, followed by PCV-13 in 2010 (8–10), which is why the local study of serotype and genotype distribution of IPD is important.

Herein, we report the genome sequences of two S. pneumoniae strains (BC18042556 and BC19010893) isolated from the bloodstream from a 22-month-old boy with meningitis and a 3-month-old girl with septic arthritis of the shoulder. Microorganisms were identified on sheep blood agar by optochin susceptibility and bile solubility testing. The MICs were determined by a Vitek 2 automated system (bioMérieux, Marcy-l’Étoile, France) and the Etest method and interpreted according to the Clinical and Laboratory Standards Institute guidelines. Genomic DNA was extracted using the automated platform NucliSENS easyMag (bioMérieux) and quantified by Qubit (Thermo Fisher, Waltham, MA). The paired-end DNA libraries were constructed with a Nextera XT kit (Illumina, San Diego, CA) according to the manufacturer’s instructions and sequenced on an Illumina MiSeq machine with 2 × 300-bp cycles.

The sequence data were evaluated using FastQC (http://www.bioinformatics.babraham.ac.uk/projects/fastqc/) and trimmed with Trim Galore (http://www.bioinformatics.babraham.ac.uk/projects/trim_galore/) using the following conditions: length, 60; quality, 20; retain_unpaired -r1 69 -r2 69; phred33). The trimmed sequence data were assembled using SPAdes v.3.9.0 (11) and assessed using QUAST v.2.3 (12). Smaller contigs (<1,000 bp) and contaminant data were excluded after analysis using Kraken v.2 (13). The STs and the antimicrobial resistance genes were predicted based on the PubMLST typing schemes in MLST (https://github.com/tseemann/mlst) and the ResFinder database (14) in ABRicate (https://github.com/tseemann/abricate) based on ≥80% coverage and 90% sequence identity. The serotypes of the strains were predicted using SeroBA (15), and the assembled contigs were annotated using the NCBI Prokaryotic Genome Annotation Pipeline (16). Default parameters were used for all software unless otherwise specified.

The genome size, serotypes, and other information are presented in Table 1. No genes encoding resistance to penicillin or cephalosporins were detected in either isolate. However, the macrolide resistance determinants *erm*(B), encoding methylases, and *mef*(A), encoding efflux pumps, were identified in the genome of BC19010893. Interestingly, although resistance to penicillin and macrolides among pediatric invasive pneumococcal isolates remains high in Qatar, a decreasing trend for the past few years has been reported, which might be related to the widespread use of PCV-7 and PCV-13 (9).

**TABLE 1:** Summary of genome statistics and relevant information

Isolate no.	No. of reads	Avg read length (bp)	Genome size (bp)	*N*_50_ (bp)	GC content (%)	Avg coverage (×)	No. of contigs	No. of CDS^*a*^	Predicted serotype	Sequence type
BC18042556	2,033,030	156	2,072,932	73,650	39.6	295	50	2,178	12F	352
BC19010893	1,735,499	146	2,084,430	55,986	39.6	260	58	2,215	9N	6359

a CDS, coding DNA sequences.

### Data availability.

This whole-genome shotgun project has been deposited in DDBJ/ENA/GenBank under the accession numbers SJSZ00000000 and SJTA00000000 and under BioProject numbers PRJNA524476 and SRA524476. The versions described in this paper are versions SJSZ01000000 and SJTA02000000.
